# Performance of an Anti-Phase Technology-Powered Microwave Ablation System on Ex Vivo Liver, Lung and Kidney: Analysis of Temperature Trend, Ablation Size and Sphericity

**DOI:** 10.1007/s00270-024-03811-z

**Published:** 2024-07-31

**Authors:** Pouya Namakshenas, Tommaso Arcaini, Benedetta Cesare, Alessandro Dorato, Elena Durante, Milena Ricci, Domiziana Santucci, Paola Saccomandi, Eliodoro Faiella

**Affiliations:** 1 Department of Mechanical Engineering, Politecnico di Milano, via Giuseppe La Masa 1, 20156 Milan, Italy; 2 Operative Research Unit of Radiology and Interventional Radiology, Fondazione Policlinico Universitario Campus Bio-Medico, via Alvaro del Portillo 200, 00128 Rome, Italy; 3 Research Unit of Radiology and Interventional Radiology, Department of Medicine and Surgery, Università Campus Bio-Medico di Roma, via Alvaro del Portillo 21, 00128 Rome, Italy

**Keywords:** Microwave ablation, Ablation size, Sphericity, Temperature distribution, Liver, Lung, Kidney

## Abstract

**Purpose:**

Investigating the performance of the new Dophi™ M150E Microwave Ablation System, in terms of temperature distribution, ablation size and shape, reproducibility.

**Materials and Methods:**

The Dophi™ M150E Microwave Ablation System was tested on ex vivo liver, lung and kidney, at 6 different settings of time, power and number of MW antennas (single antenna: 50 and 100 W at 5 and 10 min; double antenna: 75 W at 5 and 10 min). The temperature distribution was recorded by Fiber Bragg Grating sensors, placed at different distances from the antennas. The ablation axes were measured and the sphericity index was calculated.

**Results:**

The standard deviation of ablation axes was < 5 mm, except at the highest energy and time setting for the lung. A maximum temperature rise of ~ 80 °C was measured. The measured ablation axes are overall comparable with the manufacture’s values, especially at lower power and with one MW antenna (average maximum difference is 7 mm). The mean sphericity index of 0.95, 0.79 and 0.9 was obtained for the liver, lung and kidney, respectively, with a single antenna. With double antenna setup, the sphericity index was closer to 1 when 75 W for 10 min were used.

**Conclusions:**

Dophi™ M150E allows good reproducibility of ablation axes for all cases except in the lung at the highest energy level. With one antenna, an almost spherical ablation area for the liver and kidney was obtained. Using double antenna results in more homogeneous temperature distribution within the tissue compared to single antenna.

**Graphical Abstract:**

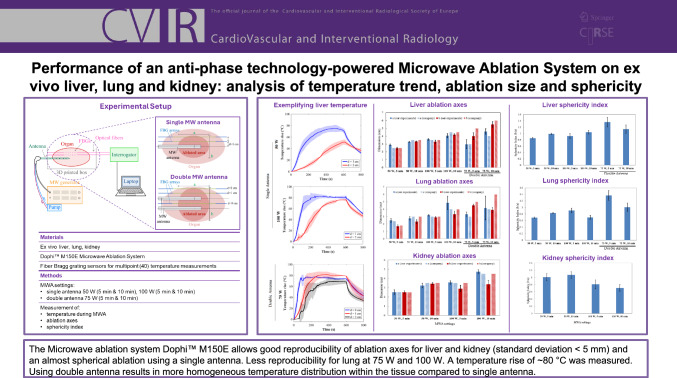

## Introduction

Microwave thermal ablation (MWA) is largely performed in interventional oncology for the treatment of liver, lung, kidney and bone tumors, as an effective alternative to surgery in patients ineligible for surgical resection, offering low complications rate, high efficacy and minimally invasive approach.

MWA allows fast increase in tissue temperature compared with RFA, resulting in a theoretically shorter procedure time and lesser impact of the heat-sink effect [1].

The key aspects of MWA towards a successful procedural outcome are a predictable ablation shape [2, 3], high reproducibility and predictability, directly linked with adequate safety margins [4].

The Dophi™ M150E Microwave Ablation System (Surgnova Healthcare Technologies) embeds several technologies which allow a controllable ablation shape: (1) the patented anti-phase technology enables the cancellation of backward microwave radiation at the end of the ablation, aiming to enhance the sphericity of ablation regions; (2) the full-antenna water cooling technology helps preventing overheat and antenna tip breakage [3]; (3) utilizing dipole antennas with floating sleeves serves dual purposes: suppressing backward currents and acting as a radiation component, leading to optimized energy transmission efficiency and enhanced specific absorption rate [4]; (4) joint point of ceramic and stainless steel shaft minimize the risk of antenna breaking. The device also includes a thermocouple-based monitoring system for measuring the temperature of the cooling fluid.

In order to fully characterize the ablation outcome of this system and to correlate it with thermal effects in the target organs, our study evaluates maximum temperature, ablation size, sphericity index and reproducibility of ablation zone in three different ex vivo animal organs (liver, lung, and kidney) using various power and time settings.

## Materials and Methods

### Organs

The organs under study were bovine liver, bovine lung, and porcine kidney. Porcine kidney was chosen due to its morphology similar to the human one and to the large availability of studies [5, 6]. Freshly excised organs were collected in a local slaughterhouse, transported to the laboratory inside an isolated ice-water-cooled container and then stored at 4 °C. Before each MWA treatment, the organs were removed from the refrigerator to achieve room temperature. A total of 9 ex vivo livers, 9 ex vivo lungs and 18 ex vivo kidneys were used in our study.

### Microwave Ablation System and Settings

The commercial device Dophi™ M150E Microwave Ablation System (Surgnova Healthcare Technologies) was used with the following settings: (1) with a single MW antenna, 50 W for 5 and 10 min, 100 W for 5 and 10 min; (2) with dual MW antennas, 75 W for 5 and 10 min. These ablation settings were selected as they are currently used for tumor treatment in the same human organs [7–10].

Dedicated 15-gauges antennas were used for liver and kidney (radiation zone length 31 mm) and for lung (radiation zone length 26 mm), as recommended by the manufacturer [11]. The cooling system consisting of a built-in pump circulated fresh water in the antennas’ shaft.

### Experimental Setup

**Fig. 1:**
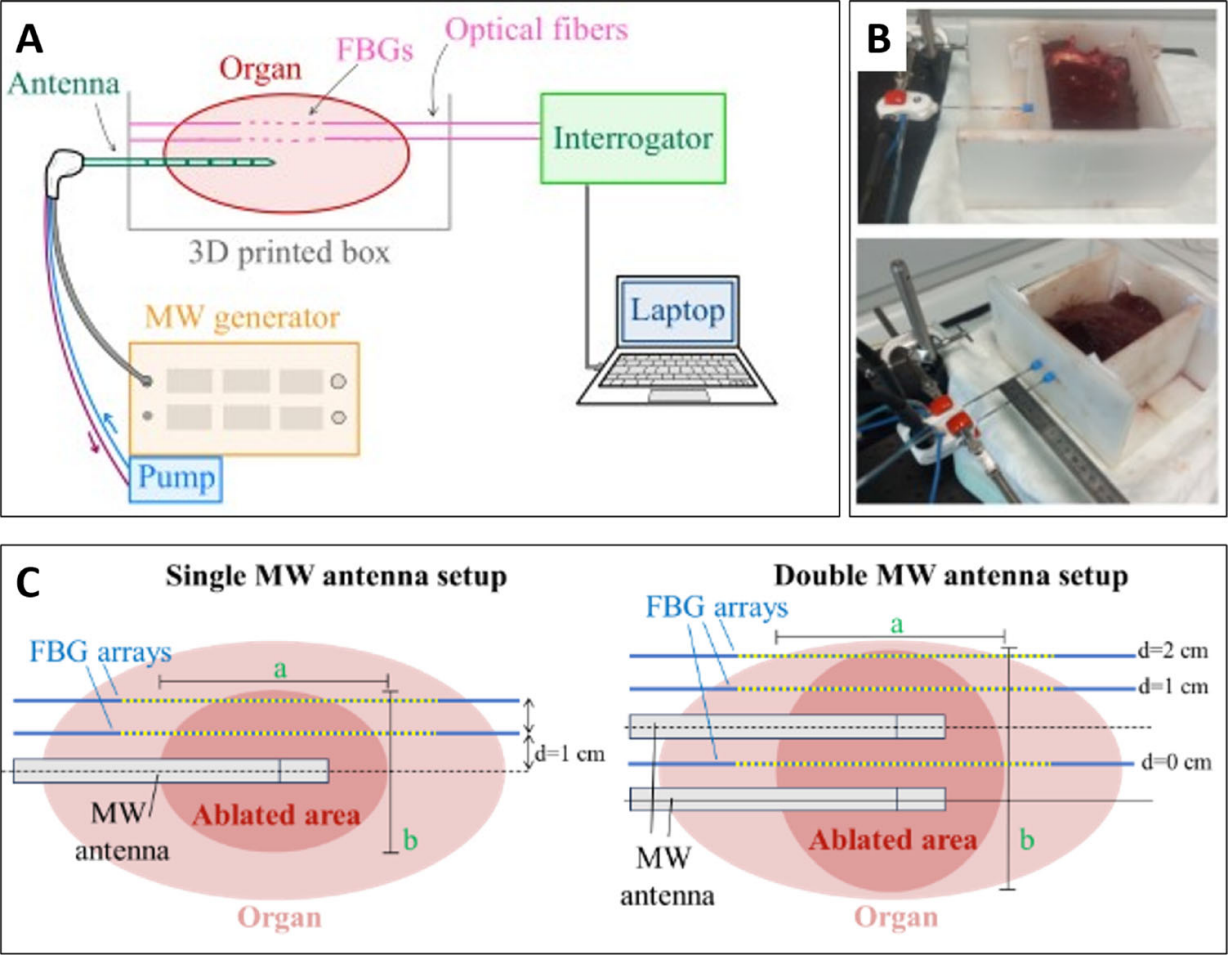
Experimental setup for ex vivo MWA.** A** Equipment used for MWA and temperature monitoring. **B** Pictures of the custom-made box made of ABS material, for both the single antenna and the double antenna configurations. **C** Placement of FBG sensors arrays in the organs and definition of the ablation axes a and b

The experimental setup consists of a custom-made box to contain the ex vivo samples, the MWA system (generator, antenna, and cooling circuit), three optical fibers with Fiber Bragg Grating (FBG) sensors, an optical interrogator, and a computer for real-time temperature monitoring during MWA (Fig. 1A). The box was created using a 3D printer; its walls included several holes with 5 mm spacing to ensure precise alignment of antennas and FBG sensors (Fig. 1B). Two configurations were implemented: single MW antenna and double MW antenna (Fig. 1B, C).

### Temperature Monitoring in Real-Time

To accurately measure the maximum temperature induced by MWA inside the organs while minimizing the number of sensors to be inserted, three arrays of FBG sensors and the Micron Optics si255 interrogation unit (Micron Optics, Atlanta, USA) were used. Each array contains 40 FBG sensors [12, 13], allowing a spatial resolution of 1.2 mm.

In the single antenna configuration (Fig. 1C), tissue temperature was measured by two FBG arrays, situated at distance (d) of 1 cm and 2 cm from the antenna. In the double antenna configuration (Fig. 1D), tissue temperature was measured by three FBG arrays, placed at d = 1 cm and d = 2 cm from one antenna, and between the two antennas (d = 0 cm).

### Experimental Protocol of Ablation and Post-Ablation Measurements

For each test, the initial temperature of the organ was 20 ± 2 °C. The tissue temperature was monitored for 10 min of ablation followed by 3.3 min of cooling. All the experiments were repeated 3 times under the same conditions. The temperature data are presented as the maximum temperature trend at 1 cm and 2 cm from the single antenna, and between antennas. These results are reported as mean temperature value ± standard deviation.

**Fig. 2:**
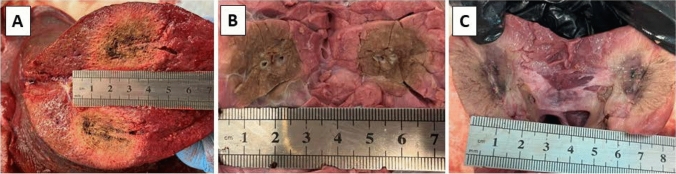
Ablation regions obtained in the liver (**A**), lung (**B**) and kidney (**C**)

**Fig. 3:**
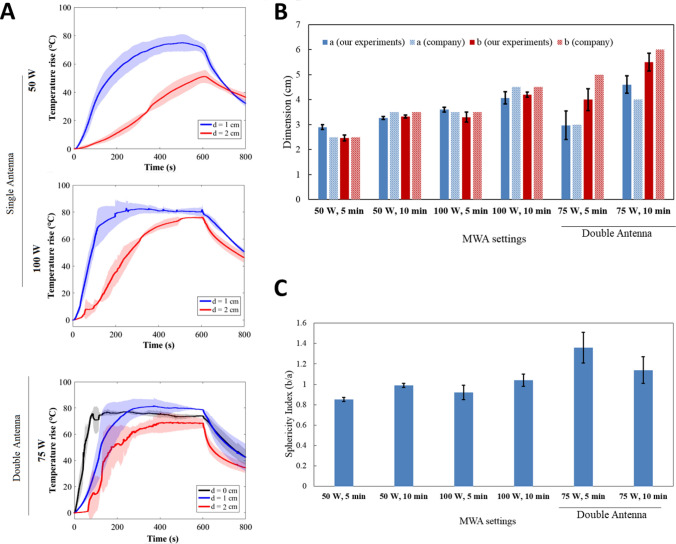
Effects of MWA on bovine liver. **A** Trends of maximum temperature at distances of 1 cm (blue) and 2 cm (red) from the MW antenna, and between the antennas (d = 0 cm, black) in the double MW antenna setting. **B** Ablation axes obtained from the ex vivo liver experiments and comparison with the company’s data at different settings. **C** Sphericity index

At the end of each MWA, the organs were cut on the plane of the antenna(s), in order to assess the area of maximum thermal damage. After visual inspection, the ablation axes (Fig. 1–3, *a* and *b*) were measured with a ruler. To ensure accurate measurement of the ablation axes within the plane containing the maximum extent, we cut the region into parallel planes (Fig. 2). These results are reported as mean axis ± standard deviation. The sphericity index was calculated as *b/a*, where *a* is parallel to the antenna axis and *b* is perpendicular to the antenna axis. The ablation areas experimentally obtained were compared with the data provided by the manufacturer.

## Results

### Bovine Liver

Results for bovine liver are shown in Fig. 3. For the single antenna setting operating for 10 min, the maximum temperature rise measured by FBG sensors is ~ 80 °C at d = 1 cm from the antenna, for both 50 W and 100 W (Fig. 3A). At d = 2 cm, the temperature rise reaches 50 °C at 50 W and 75 °C at 100 W. When two antennas are used, the maximum temperature rise between antennas (d = 0 cm) and at 1 and 2 cm distance is more homogeneous compared to single antenna and varies between 60 and 80 °C.

The analysis of the ablation zone (Fig. 3B) obtained with single antenna settings at 50 W and 100 W shows a small standard deviation (< 3 mm) on *a* and *b*, and results are comparable to the data provided by the company. However, for the dual-antenna configuration, a difference up to 1 cm for the *b* axis after 5 min MWA and a higher standard deviation (up to 5 mm) are reported (Table 1).

**Table 1:** Post MWA analysis in liver, lung and kidney: ablation axes, comparison with data provided by the manufactures and sphericity index

MW setting	a [cm]	b [cm]	a-a_company_ [cm]	b-b_company_ [cm]	Sphericity index, b/a
*LIVER*					
50 W 5 min, Single antenna	2.9 ± 0.1	2.5 ± 0.1	0.4	0	0.85
50 W 10 min, Single antenna	3.3 ± 0.1	3.3 ± 0.1	− 0.2	− 0.2	1
100 W 5 min, Single antenna	3.6 ± 0.1	3.3 ± 0.2	0.1	− 0.2	0.92
100 W 10 min, Single antenna	4.1 ± 0.2	4.2 ± 0.1	-0.4	− 0.3	1.02
75 W 5 min, Double antenna	3.0 ± 0.6	4.0 ± 0.4	0	− 1	1.36
75 W 10 min, Double antenna	4.6 ± 0.4	5.5 ± 0.4	0.6	− 0.5	1.14
Mean difference: experiments vs manufacturer [cm]	–	–	0.3	0.4	–
*LUNG*					
50 W 5 min, Single antenna	2.5 ± 0.3	1.7 ± 0.2	0	− 0.1	0.68
50 W 10 min, Single antenna	2.8 ± 0.2	2.3 ± 0.3	-0.3	− 0.7	0.82
100 W 5 min, Single antenna	3.2 ± 0.1	2.9 ± 0.1	0.2	− 0.1	0.91
100 W 10 min, Single antenna	4.9 ± 0.9	3.3 ± 0.4	0.9	− 0.7	0.67
75 W 5 min, Double antenna	3.3 ± 0.3	4.5 ± 0.2	0.3	0.5	1.36
75 W 10 min, Double antenna	4.2 ± 1.6	3.9 ± 0.4	0.2	2.1	0.93
Mean difference: experiments vs manufacturer [cm]	–	–	0.3	0.7	–
*KIDNEY*					
50 W 5 min, Single antenna	2.5 ± 0.3	2.5 ± 0.2	0	0	1
50 W 10 min, Single antenna	3.1 ± 0.1	3.3 ± 0.2	− 0.4	− 0.2	1.06
100 W 5 min, Single antenna	3.6 ± 0.2	2.9 ± 0.4	0.1	− 0.6	0.81
100 W 10 min, Single antenna	4.9 ± 0.2	3.4 ± 0.2	0.4	− 1.1	0.73
Mean difference: experiments vs manufacturer [cm]	–	–	0.2	0.5	–

With single antenna, the sphericity index is close to 1 for 50 W and 100 W at 10 min, while it ranges between 0.85 and 0.9 for 5 min MWA. The double antenna setting allows obtaining a sphericity index bigger than 1, especially for 5 min MWA (Fig. 3C).

### Bovine Lung

**Fig. 4:**
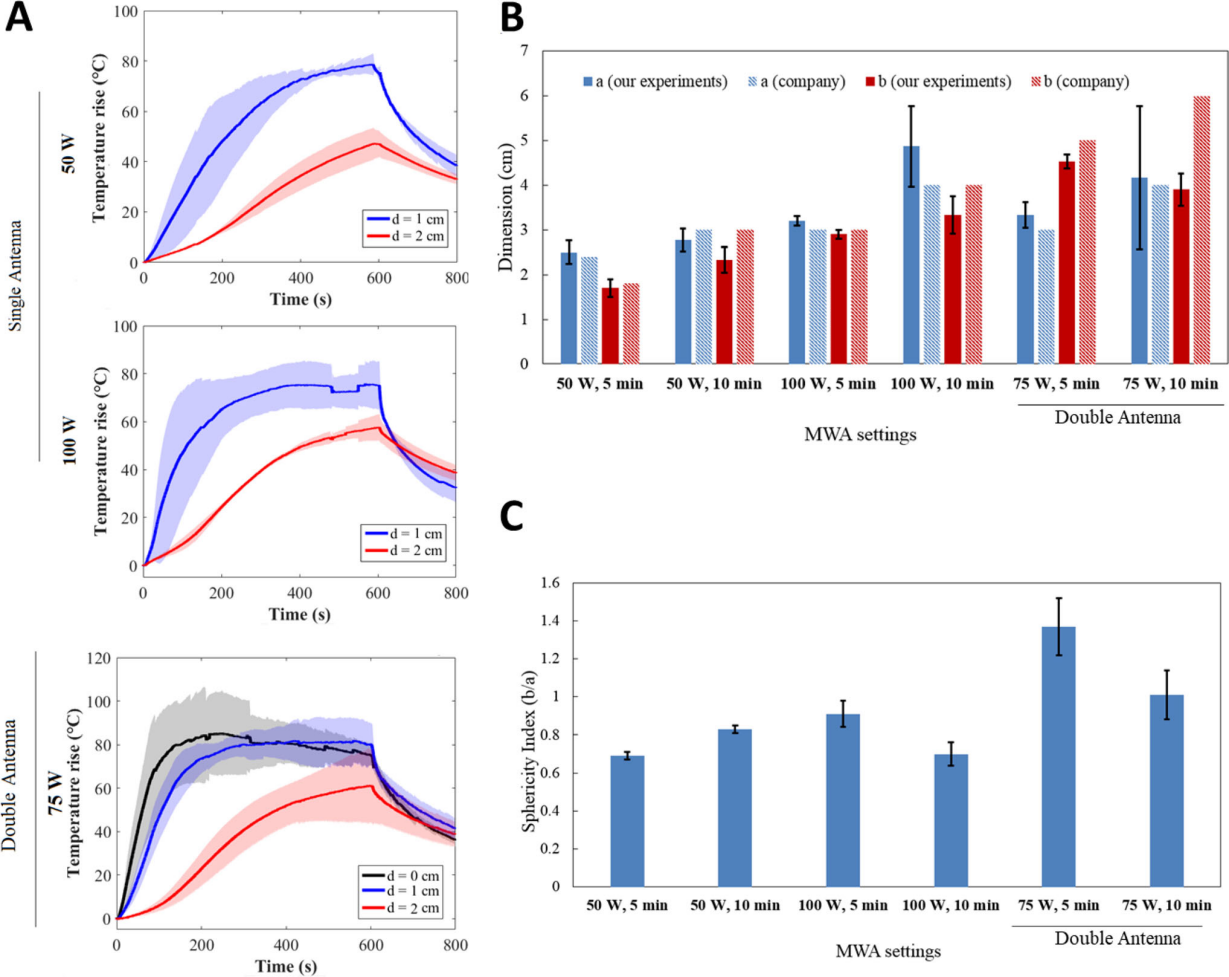
Effects of MWA on bovine lung. **A** Trends of maximum temperature at distances of 1 cm (blue) and 2 cm (red) from the MW antenna, and between the antennas (d = 0 cm, black) in the double MW antenna setting. **B** Ablation axes obtained from the ex vivo lung experiments and comparison with the company’s data at different settings. **C** Sphericity index

Results for bovine lung are presented in Fig. 4. For the single antenna settings, the maximum temperature rise measured by FBG sensors reaches about 80 °C at d = 1 cm from the antenna, for both 50 W and 100 W after 10 min MWA (Fig. 4A). At 100 W, the maximum temperature rise varies between 50 and 60 °C at d = 2 cm from the antenna. For the double antenna configuration, the maximum temperature rises measured at d = 0 cm and d = 1 cm are similar, with a plateau at ~ 80 °C which lasts at least 300 s. Conversely, the trend measured by the sensor at d = 2 cm is similar to the trends at d = 2 cm observed for the single antenna setting.

The analysis of the dimensions of the ablation area (Fig. 4B) obtained with single antenna settings at 50 W and double antenna at 75 W-5 min shows a standard deviation < 3 mm on both *a* and *b.* The standard deviation is 1 mm on both *a* and *b* for the setting 100 W-5 min, whereas it increases for all the other settings. The results are overall comparable to the data provided by the company, with the exception of the settings at 10 min, at which we register a maximum difference of 8.7 mm for the *a* axis at 100 W (Table 1).

The sphericity index (Fig. 4C) is close to 1 for double antenna at 75 W-10 min, thus obtaining a spherical shape. It is 1.4 when 75 W is delivered by two antennas for 5 min, and it is < 1 for all the other settings.

### Porcine Kidney

**Fig. 5:**
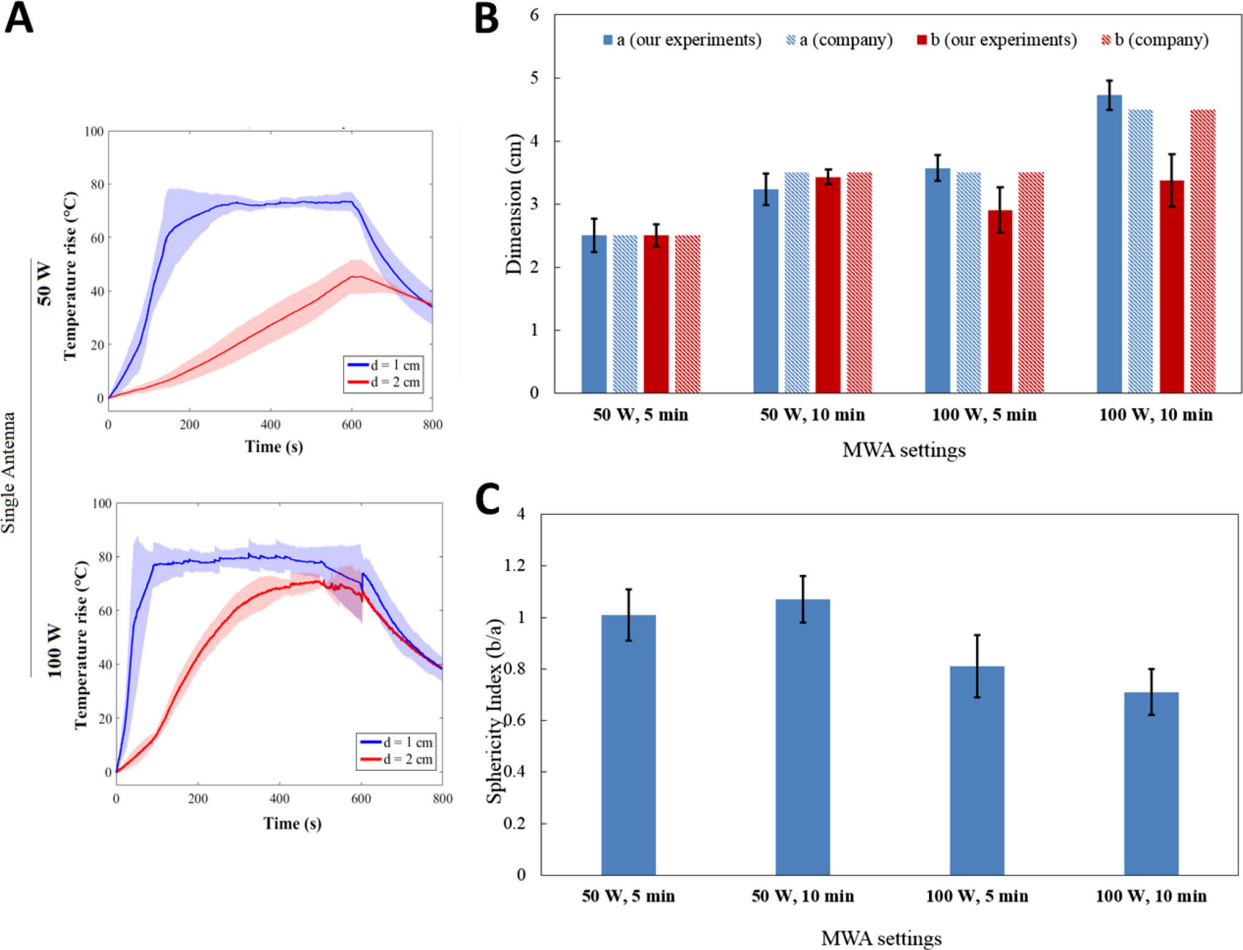
Effects of MWA on porcine kidney. **A** Trends of maximum temperature at distances of 1 cm (blue) and 2 cm (red) from the MW antenna. **B** Ablation axes obtained from the ex vivo kidney experiments and comparison with the company’s data at different settings. **C** Sphericity index

Results for porcine kidneys are shown in Fig. 5. Due to the small size of porcine kidneys, it was not possible to use the double antenna configuration. The temperature rise profiles shown in Fig. 5A are similar to the ones obtained for the bovine liver: the maximum temperature rise at d = 1 cm from the antenna is ~ 75 °C for 50 W and ~ 80 °C for 100 W. At d = 2 cm, the temperature rise reaches ~ 50 °C at 50 W and 75 °C at 100 W.

As the ablation dimensions for the kidney were not provided by the company, the data for the liver were used as a reference, since the parenchyma of both organs is epithelial tissue. According to Fig. 5B, our measurements are comparable to the company’s values for 50 W and for 100 W-5 min. However, at 100 W and 10 min, we observed a deviation of 1.1 cm from the company data for the axis perpendicular to the antenna. The measurements show a good reproducibility, as the standard deviation is < 3 mm in most cases (Table 1).

Spherical ablation areas are obtained when the kidney is treated with 50 W (the sphericity index is 1 after 5 and 10 min MWA), whereas an elliptical shape is obtained with 100 W.

## Discussion and Conclusions

The performance of the Dophi™ M150E Microwave Ablation System is satisfactorily predictable for the organs under study.

The temperature trends are consistent with the applied MW settings. For liver and kidney, the temperature rise in time (Fig. 3A and 5A) and the slopes of the temperature curves are very similar, proving that it is a reasonable choice to use the settings defined for the liver also for treating kidney [14], as these organs are characterized by similar thermal properties [15–17]. Indeed, the thermal effect and the resulting size and shape of ablation region depends on the heat propagation inside the tissues, which is described by the idiosyncratic thermophysical properties. Lastly, the three independent experiments at the same conditions provide a good reproducibility, as witnessed by an average standard deviation < 10% on all the curves.

In terms of ablation areas, both liver and kidney present high reproducibility and correspondence to the data of the manufacturer when 50 W is used. Increasing the power values to 100 W leads to a lower reproducibility (increased values of the standard deviation), and bigger difference with the data provided by the manufacturer (Figs. 3B–C, 5B–C and Table 1). For the kidney, in particular, at 100 W, the measured *a* axis is comparable with the manufacturer data, whereas the measured *b* axis is smaller, with a difference of 0.6 cm and 1.1. cm after 5 min and 10 min of MWA, respectively. The possible cause of this low reproducibility is the small size of the porcine kidney, which poses a challenge in performing complete ablations in the interior portion of the organ without affecting its surface. Conversely, it results to be a useful model to study the MWA effects when the power of 50 W is used.

The sphericity index, which is a measure of the spherical shape of the ablation (sphericity index = 1 corresponds to spherical ablated area), has different trends for liver and kidney. For the liver, it ranges from 0.85 to 1, using a single antenna at 50 W and 100 W. These results agree with outcomes obtained with Emprint ablation system (Covidien/Medtronic, Minneapolis, USA), which holds similar technologies of Dophi™ M150E Microwave Ablation System [2]. In ex vivo experiments conducted on porcine livers using the Emprint Ablation System, the mean sphericity index was found to be 0.87, whereas for the HS Amica-Gen device (HS AMICA PROBE, HS Hospital Service, Aprilia, Italy) and NeuWave Certus Microwave Ablation System (NeuWave Medical, Madison, Wisconsin), it was 0.59 [4] and 0.75, respectively [2, 4, 18]. A multicentre retrospective study, conducted across two hospitals in France and one in the USA, included MWA of liver tumors, comprising 65 metastases and 22 hepatocellular carcinomas, using the Dophi™ M150E MWA system (HDTECH, Lorient, France), and reported a mean sphericity index of the ablation zone as 0.78 ± 0.14 [4].

For the kidney, sphericity index was 1 delivering 50 W with one antenna for both 5 and 10 min, while it ranges between 0.7 and 0.8 for 100 W.

Regarding bovine lung, the maximum temperature rise is similar to what obtained for liver and kidney, but the different temperature rises measured at d = 2 cm (i.e., 45 °C at 50 W, 55 °C at 100 W and 60 °C at 75 W-dual antenna) suggest a peculiar temperature trend (Fig. 3A). This might be ascribed to the specific antenna geometry which is recommended for the lung (active length of 26 mm), and to the tissue characteristics. The standard deviation on the ablation axes of the lung (Fig. 4B) is overall bigger than the one obtained for the liver (Fig. 3B), especially on *a* axis (which is parallel to the path of the antenna). This deviation might be caused by the presence of several bronchi inside the lungs, which contributes to a heat sink effect and lower ablation reproducibility when high power settings are used [3]. However, in in vivo scenarios, the ablation regions are smaller than the ones obtained in ex vivo models with the same MW settings [3].

The sphericity index obtained in the lung is < 1 for most settings, except for double antenna at 75 W-5 min. For both liver and lung, this setting allows a sphericity index = 1.36, with an ablation area elliptical, larger in the direction perpendicular to the antenna’s axis (*b* axis) than longer (*a* axis). MWA performed with more than one antenna allows a tailored ablation area, reproducing the shape and size of the lesion with an adequate safe margin [19, 20]. This aspect is crucial to establish the efficacy of MWA, as it has been observed that tumor size increases the risk of local recurrence [21, 22].

The target to reach for every ablation treatment is A0 (Ablation Zero), which means no pathological tissue residual. To achieve  a complete tumor eradication the operator must consider the ablative margin, which is the region ablated beyond the tumor’s perimeter; this margin, ideally, should measure 0.5–1.0 cm in its smallest width, depending on tumor histological type [23].

Considering the same tissue features, the technical parameters that can affect the results are predictability, reproducibility and sphericity index.

Using Dophi™ M150E Microwave Ablation System, the comparison of the experimental ablation axes and the values provided by the manufacturer provides good results especially at lower power and with one antenna (average difference ranges between 2 and 7 mm, Table 1). Using the double antenna setting decreases the performance in terms of predictability of the ablation axes obtained in the liver and in the lung (Fig. 3B, 4B), and provides sphericity index > 1 (except for the lung treated at 75 W-10 min).

However, our results are valid for ex vivo healthy organs (for direct comparison with manufacturer’s indications): data obtained in in vivo scenarios might reveal increased variation compared to company’s values, because of heterogeneity in tumor type.

Variations in heating during longer treatments may be related to thermal conduction and convection of heterogeneous tissues, and suggest preferring short, high-power ablations over long, low-power ablations for the sake of reproducibility [4].

There are some limiting aspects of the study that require a deeper analysis: ablations were carried out in unperfused healthy porcine organs, thus the ablation systems used in this work can perform differently when applied in clinical scenarios. The company provides several settings and needles, but we focused this initial analysis on the most used settings (following the recommendation from radiology experts) and one single needle type for each organ. In future, it will be useful to study the performance of the system at several combinations in power and ablation time, and with different needles.

To conclude, the results of our ex vivo experience with Dophi™ M150E can affect the clinical practice and impact on ablative treatments outcome. In the future, more power-time combinations would be assessed on target organs, and dedicated imaging approaches will be used to evaluate the ablated volumes.
