# Correction to: Performance of an Anti-Phase Technology-Powered Microwave Ablation System on Ex Vivo Liver, Lung and Kidney: Analysis of Temperature Trend, Ablation Size and Sphericity

**DOI:** 10.1007/s00270-025-03970-7

**Published:** 2025-01-28

**Authors:** Pouya Namakshenas, Tommaso Arcaini, Benedetta Cesare, Alessandro Dorato, Elena Durante, Milena Ricci, Domiziana Santucci, Paola Saccomandi, Eliodoro Faiella

**Affiliations:** 1 Department of Mechanical Engineering, Politecnico di Milano, via Giuseppe La Masa 1, 20156 Milan, Italy; 2 Operative Research Unit of Radiology and Interventional Radiology, Fondazione Policlinico Universitario Campus Bio-Medico, via Alvaro del Portillo 200, 00128 Rome, Italy; 3 Research Unit of Radiology and Interventional Radiology, Department of Medicine and Surgery, Università Campus Bio-Medico di Roma, via Alvaro del Portillo 21, 00128 Rome, Italy

**Correction to: Cardiovasc Intervent Radiol (2024) 47:1392–1401** 10.1007/s00270-024-03811-z

The given names and family names of the article’s authors were listed in the wrong order in the published article.

The correct order, given names first followed by family names, is listed below:

Pouya Namakshenas

Tommaso Arcaini

Benedetta Cesare

Alessandro Dorato

Elena Durante

Milena Ricci

Domiziana Santucci

Paola Saccomandi

Eliodoro Faiella

The original article has been corrected.

